# Involving Children Under 5 years in a Hand Hygiene Intervention Study at Preschools in South Africa

**DOI:** 10.1177/11786302261417911

**Published:** 2026-05-08

**Authors:** Samantha Louise Lange, Tobias George Barnard, Nisha Naicker

**Affiliations:** 1 Water and Health Research Centre, Faculty of Health Sciences, University of Johannesburg, South Africa; 2 Environmental Health Department, Faculty of Health Sciences, University of Johannesburg, South Africa; 3 Epidemiology and Surveillance, National Institute for Occupational Health, National Health Laboratory Services, Braamfontein, South Africa

**Keywords:** hand hygiene, reflexivity, involving children, children’s voices, micro-ethical moment

## Abstract

**Background:**

Involving children in research may include opportunities for their participation in assisting in the development of appropriate data collection tools, providing ideas for interventions and even collecting data themselves. Using a reflexive approach to research by not having preconceived ideas, and recognising that children’s participation can play an important role in improving the quality of the research. This paper aims to show how applying reflexivity and noting children’s actions during research improved the ethical and methodological quality of a study, providing a roadmap for future researchers to follow.

**Methodology:**

An action-based approach, using the actions and reactions of the study participants, was applied during a hand hygiene intervention conducted at preschools in South Africa (N = 17). Data was collected from 19 caregivers, 191 parents and 160 children aged 4 to 5 years using various data collection tools. During the pilot and main study, the researcher made use of field notes to apply a measure of reflexivity and adaptability during the data collection and intervention stages.

**Results:**

Ethical considerations and methods were improved through the actions of the child participants. Children reacted with discomfort and trepidation to the swabbing of hands and use of unfamiliar research assistants to collect data, resulting in a change in the data collection methods. Actions of children during the observation phase resulted in additional data being collected to determine any significance between activities and HH moments. Handwashing was changed to include physical hand movement guidance, as 18.5% of children were unable to complete handwashing steps without assistance.

**Conclusion:**

Researchers should apply reflexivity and adaptability when involving children, ethically prioritising children’s needs and interpreting children’s voices, with the child’s best interest and comfort being paramount. Research protocols should be continuously adapted and revised based on participants’ reactions during the study to improve the quality of research processes.

## Plain language summary

Involving children in a hand hygiene intervention study

The aim and purpose of this paper was to highlight the important role children can play in the research process. Previously, research was conducted “on” children, as opposed to “with” them and this paper aims to show how children shaped the data collection, interventions and ethics of the study. The study formed part of a larger study to investigate whether a hand hygiene intervention could reduce hand hygiene-related diseases. Diarrhoeal diseases are one of the leading causes of death in children under 5 years and can be prevented or reduced through hand hygiene interventions such as proper hand washing at the correct times. An action-based approach was applied to an intervention study, observing children’s actions and reaction to the various aspects of the study. The intervention consisted of teaching children to wash hands using GloGerm^®^ and a “Magic Box.” Data was collected using questionnaires, observations, inspections and logging of hand hygiene-related illnesses and the researcher made field notes of the actions of the participants throughout the study, which are reported in this paper. The findings showed how, by listening to children and observing their actions and reactions to various aspects of the research process, researchers can reflect on these actions and adjust the research process to improve methodologies and ethical considerations. Researchers can relate to the findings in this paper and apply similar reflexive principles to their own research, especially when the research participants are children. These findings can also influence research policies to apply reflexivity to future research protocols and studies.

## Introduction

United Nations Convention of the Rights of the Child (1989) declared that children had the right to express their views and opinions freely on all matters that affected them. Involving children in research and researching with them as opposed to on them was previously discouraged as children were not deemed a credible source of information. Sarkadi et al^1^ further explained that research traditionally was about children, but that research with children and by children are 2 concepts which provide data from study subjects who are no longer seen as passive, but through their agency, can contribute positively within the research setting.^1^ Not all studies are designed to include their study subjects in a participatory way, there are, however several ways that study participants and more importantly, children, can contribute towards the outcomes of a study. These can include assisting in developing data collection tools, such as making suggestions on the types of questions to be asked; what materials to use and which research topics are suggested by the participating community.

Listening to children’s voices and identifying what can be classified as children’s voices can allow the researcher to make adjustments to the research project in a way that allows children to feel comfortable participating. Young children often cannot verbalise their needs or concerns but may express themselves through laughing, crying, smiling or gesticulating.^2^ Furthermore, involving children in research for example, by assisting with the design of an intervention, either through active participation, such as assisting to develop a hand hygiene toy^3^ or allowing them to help develop a handwashing robot^4^; or researchers taking note their behaviour during the initial phases or pilot study and implementing these findings, can assist in the success of the research outcomes.

Having a reflexive approach to research with children by not creating preconceived ideas and creating opportunities for fun and empowerment in the research process, as well as recognising children’s participation, should be acknowledged.^1^ Studies have shown that asking questions is an integral part of children’s learning, where a study in a United States preschool, during open playtime, assessed children’s question-asking and determined that children would determine “whom” to ask “What” depending on who they felt to be more knowledgeable, asking more questions of adults than their peers.^5^

Applying various types of hand hygiene (HH) interventions in order to improve hand hygiene and reduce the burden of HH-related diseases has become an acceptable method of achieving these goals,^6,7^ with a systematic review reporting that promotion of hand washing could reduce diarrhoeal illnesses by up to 30%.^8^ Diarrhoea accounts for over 1 million deaths of children under 5 years worldwide^9^ and hand washing with soap can reduce the risk of diarrhoeal disease by 42% to 47%.^10^

HH interventions, often with hand washing demonstrations in one form or another, have successfully been implemented to kerb the spread of infectious diseases and improve health outcomes.^8,10,11^ Children in particular are at risk regarding HH-related and HH-preventable diseases such as diarrhoea, as they are considered to be a vulnerable group due to their still-developing immune system and their propensity for using hands and mouths to explore the world around them in a tactile manner.

Children need to be motivated to learn, and beliefs such as “Do I belong here?” or “Does my teacher understand me?” or “Can I trust my teachers?” need to be acknowledged to facilitate the motivation to learn.^12^ Similarly, researchers need to understand that children may think similarly in relation to the researcher, and allaying these concerns regarding trust, understanding, and belonging can benefit the outcome of the study.

Over time, researchers have employed various strategies to implement HH interventions amongst children. These strategies include the use of novelty soaps, where toys are inserted into the soap and children were 4 times more likely to wash their hands in order to reach the toy^13^; making use of a Glo-Yo, a type of yo-yo with HH messages, a lotion dispenser for Glo-germ^®^ and a UV light, increasing HH awareness by 24.4%^14^ and using a clown to demonstrate handwashing thereby reducing bacterial colonisation on children’s hands by 50%.^15^ The success of these interventions have been attributed to presenting interventions in an entertaining and interesting way, similar to successes found in applying a learning-through-play approach to motivate children to read.^16^

Although the most common way of involving children in research is to establish an advisory group of children to assist in the planning and implementation^17^ this study made use of observations made by the researcher during various stages of the study which allowed changes to be made. Studies using children under 5 years as active researchers or co-designers are relatively scarce; however some studies reported using children in this age group to refine aspects of the study.^18^ Observing children’s reactions and behaviour during a study and making those adjustments and refinements to a study can potentially be used as a passive co-design instrument. This paper aims to provide a narrative account of experiences while working with children during a hand hygiene intervention conducted at preschools in South Africa,^19^ as well as highlighting how children helped shape aspects of data collection and the intervention in the study, using an action research approach to capture the various reactions and behaviours of the children.

## Methodology

### Sampling

An intervention study with an experimental design was conducted at 17 schools in the Kempton Park area of Gauteng, South Africa, collecting data from children aged 4 to 5 years (N = 160) and their parents (N = 191) and preschool caregivers (N = 19) from February to December 2019. The schools were in urban areas, compliant with the structural and legislative requirements of registered preschools,^20^ with similar water supplies, toilets and handwashing facilities. Once randomly selected, the schools were blindly allocated to an intervention group (IG) and a control group (CG).

#### Data Collection

Data was collected pre- and post-intervention in both groups using questionnaires to caregivers at the preschools (n = 19); household questionnaires for parents (n = 191); bacterial hand samples for children; hygiene inspections of the preschools (n = 17); disease registers kept by preschool caregivers and observations of children and caregivers hand hygiene practices while in the classroom (n = 346). Prior to commencement of the research project, a pilot project was initiated at 9 preschools (IG n = 7 and CG n = 5) to ensure that data collection tools would be sufficient and that the intervention was applicable.^19^ Some of the results discussed in this paper were obtained during the pilot study and will be indicated as such.

The researcher applied an action-based approach throughout the intervention study. At each of the various data collection stages in both the pilot and main study the researcher created field notes collecting information on the following:

- Reaction of children to the hand swab collection phase
- Actions of individual children during the hand hygiene opportunity observation phase
- Actions and compliance of children during the hand-washing interactive demonstration phase
- Actions and attitudes of children towards the researcher after the intervention during post-intervention data collections

#### Data Analysis

The action-based data collected were divided into themes depending on the action. If there was a negative reaction to the hand swabbing it was recorded. An example of this is if a child displayed hesitance or discomfort, it would be noted. During the hand washing phase, actions of children not able to complete the hand washing motions without assistance were noted, as well as their gender. Positive actions and attitudes during subsequent visits following the intervention were noted. These themes were then quantified and presented numerically or as frequencies where possible.

The field notes were used to make adjustments to the data collection project and to add additional depth to the findings of the study through analysis. The additional data collected during the HH opportunity intervention phase was captured on an Excel spreadsheet and although it did not form part of the objectives of the original study the results were analysed for frequency pre-and post-intervention and Chi-square analysis was applied to determine any significant increase or decrease of children’s actions pre-and post-intervention. Data was also analysed using the Pearson correlation coefficient to determine whether the children’s activities which they were undertaking at the time of the observation had any significant relationship to their hand hygiene opportunities observed.

Micro-ethical moments during the procurement of ethical consent and during the research process were documented by the researcher and added to the field notes. The micro-ethical moments for this study can be seen as any time a child’s needs, comfort, or well-being may be affected in the process of collecting data for the study. These moments were logged and included children enrolled in the study and those who were not but were exposed to various aspects of the study by virtue of being present during data collection.

### Intervention

**Figure 1.**
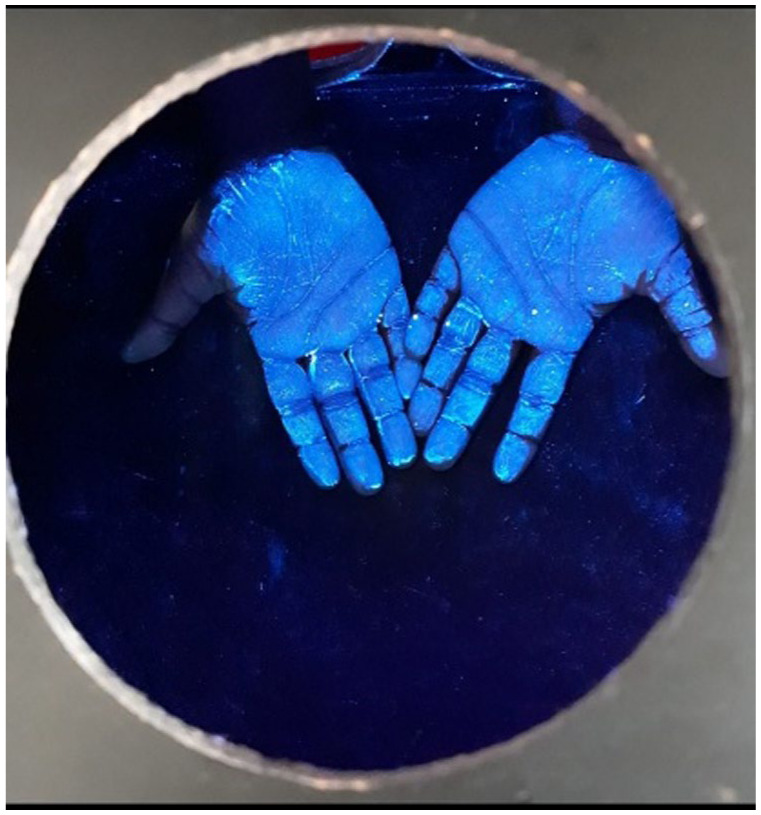
Glo Germ^®^ on hands.

**Figure 2.**
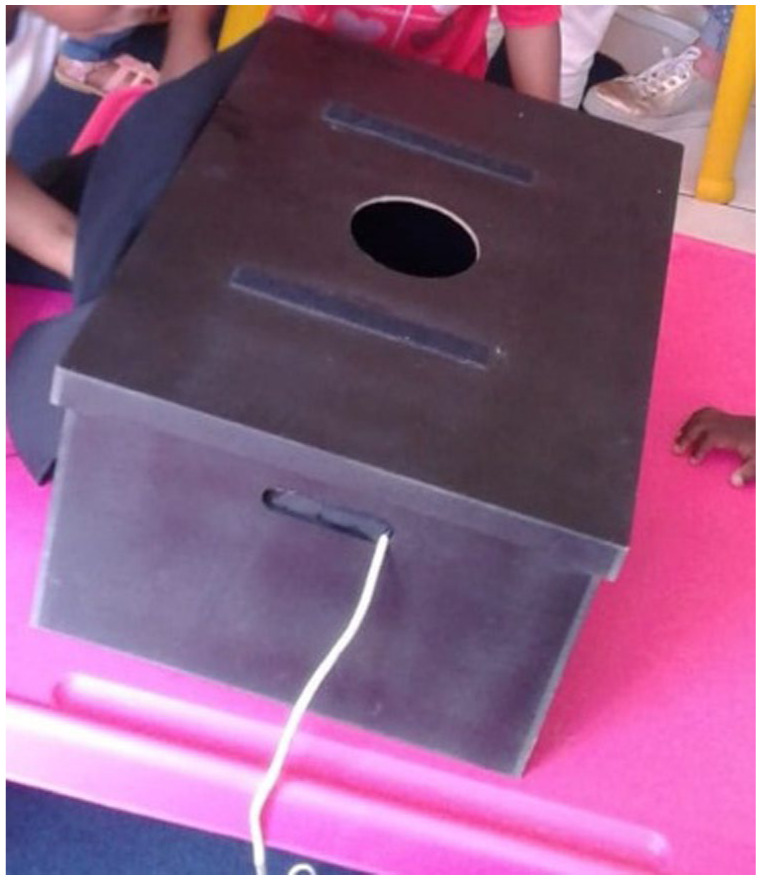
“Magic box.”

The intervention consisted of a handwashing activity for the preschool children where all children (N = 346) in the identified classes were exposed to the activity, regardless of their being enrolled in the study. The World Health Organisation (WHO) handwashing method^21^ was demonstrated to the children. Children then had Glo Germ^®^ applied to their hands and placed their hands within a “Magic Box” to see the Glo Germ^®^ on their hands (Figure 1). The “Magic Box” consisted of a wooden box fitted with an ultraviolet light with a side opening for children to slide their hands into and a viewing hole on top of the box for children to observe their hands glowing under the ultraviolet light (Figure 2). Children washed their hands using the WHO method, observed by the researcher who guided them in the different steps and corrected incorrect movements. Children then observed their hands again to see if the Glo Germ^®^ had been effectively washed off. A 21-day sticker chart, with stickers, was left in the classroom allowing children to stick stickers onto the chart for every day that they adhered to proper handwashing. An additional intervention included leaving a handwashing poster in the classroom and sending handwashing messages and information to parents for 3 weeks.

During the data collection and intervention period, the researcher made field notes of various points of interest observed during this time, which will be included in accounting for how children indirectly contributed towards shaping the methodology and results of the research project.

## Results

Having children participate in research can influence a study in several ways. This section will explore how the children, as study participants, influenced data collection methods and the intervention, based on the researcher’s field notes. The results will also indicate the ethical steps taken and changes made in the approach to include children in the study in an ethically responsible manner.

### Reaction of Children to the Hand Swab Collection Phase

There were several aspects of the study where children directly influenced aspects of data collection. The first of these was the bacterial hand samples taken from the children enrolled in the study. During the pilot study, bacterial hand samples were taken making use of Transsystem^®^ Sterile Transport Swab to determine the presence of Escherichia coli, on the children’s hands as an indicator of poor hand hygiene. The swab was rolled over the palms, between the fingers and around the fingernails of the dominant hand of each child in situ (N = 71), without handwashing or any other procedure before swabbing. The samples were then transported to a laboratory for analysis. This was repeated post-intervention (N = 68). Several children raised concerns that the swab was a type of medical intervention and associated with something similar to an injection. The researcher explained the purpose of the swabs and the results they were going to test for. Bacterial hand samples were collected using a “Bag-wash” method for the main study to allay any potential fear the children may have had regarding the sample method.

Additionally, the researcher conducted swabbing at one of the selected preschools with the assistance of 2 male students who had not previously accompanied the female researcher. The students (who were being used as fieldworkers as part of their work integrated learning) were met with resistance and a small measure of fear, possibly attributed to “stranger danger,” from several children when they attempted to take the hand swab samples. The researcher was then faced with the ethical dilemma of needing to collect data while respecting the dissent of the children. Once the researcher intervened and took the samples the children relaxed and were co-operative. As preschools are mainly the domain of female caregivers (in this study 100% of preschool caregivers were female) the decision was made that only females would assist the researcher and that, where possible the researcher would take the samples herself.

Analysis of the swabs both pre- and post-intervention did not show *E. coli* growth although in some instances there was evidence of *Proteus mirabilis* which can be found in soil^22^ and the presence was attributed to the children playing outside in the dirt on the playground. As no *E. coli* was found, even though some children’s hands were visibly dirty it was decided to replace hand swab samples with “Bag-wash” samples which would be analysed using flow cytometry.^23^ Changing the collection method provided results that could be quantified as live intact cells and total bacterial count to determine handwashing compliance in the children. Additionally, switching to “Bag-wash” samples was found to be more acceptable to the children and perceived by them to be a less “medical” approach.

### Actions of Individual Children During the Hand Hygiene Opportunity Observation Phase

A further aspect where children’s behaviours influenced the data collection process was during the HH opportunity observation phase. Preschool classes were observed for 20 minutes, and all hand hygiene opportunities were noted with a corresponding note if the caregiver or child acted on the opportunity. During the pilot study observation, the researcher noted that children placed fingers or objects in their hands quite often and tended to do so more often depending on the activity. Although this was not part of the observation protocol, additional notes were taken during the main study to determine how often children placed hands or objects in their mouths and whether this could be linked to a specific activity. During the observation phase of 17 classes involved in the study, activities that the children were engaged in at the time were placed into 5 main categories namely: Classwork which included writing, matching shapes, counting, crafts and painting; colouring with wooden pencils included writing or colouring specifically making use of wooden pencils; eating midmorning snacks and birthday snacks; story time sitting at chairs or on the floor being read to by the caregiver; and indoor playtime included building with blocks, Lego^®^, playing with dolls and cars. Table 1 displays the results as numbers and percentages of children completing various actions, with means and standard deviations for the Pearson Chi-square analysis, as well as the statistical significance (*P* < .05) of the number of times hands and objects were placed in mouths both pre- and post-intervention. Analysis did not reveal any significant results based on the activity linked to the hands or objects in the mouth.

**Table 1.** Activities, Children and HH Opportunities.

Activity^a^	IG pre-intervention no. children	IG post-intervention no. children	CG pre-intervention no. children	CG post-intervention no. children	Mean pre-intervention (fingers in mouth)	Mean post-intervention (fingers in mouth)	Mean pre-intervention (objects in mouth)	Mean post-intervention (objects in mouth)
Classwork	115 (59.0%)	64 (32.0%)	70 (47.0%)	55 (50.0%)	10.5 (SD4.17)	8.67 (SD6.8)	3.1 (SD4.1)	2.67 (SD1.2)
Colouring	30 (15.0%)	29 (14.0%)	60 (40.0%)	19 (17.0%)	12.6 (SD3.5)	8.5 (SD5.0)	8.4 (SD4.1)	6 (SD2.1)
Eating	30 (15.0%)	30 (15.0%)	-	-	10.5 (SD0.7)	-	3.5 (SD4.9)	-
Storytime	22 (11.0%)	50 (25.0%)	19 (13.0%)	13 (12.0%)	22 (SD8.4)	12 (SD1.8)	3.5 (SD3.5)	2.5 (SD3.6)
Indoor play	-	29 (14.0%)	-	24 (21.0%)	-	7.8 (SD2.8)	-	1.5 (SD2.3)
Total no. children	197	202	149	111				
*p*-Value*					0.210	0.455	0.337	0.486

a Activities: 1 = Classwork which included writing, matching shapes, counting, crafts and painting; 2 = Colouring with wooden pencils included writing or colouring specifically making use of wooden pencils; 3 = Eating midmorning snacks and birthday snacks; 4 = Story time sitting at chairs or on the floor being read to by the caregiver; 5 = Indoor playtime included building with blocks, Lego^®^, playing with dolls and cars.

* Significant *P*-value < .05.

### Actions and Compliance of Children During the Hand-Washing Interactive Demonstration Phase

The WHO method of handwashing was demonstrated for children to mimic during the intervention phase of the research project. During the intervention when children were asked to apply this method, the researcher noted that there were 8 female and 28 male children (18.5%) who were not able to perform this hand-washing sequence without the assistance of the researcher placing their hands in the correct position, indicating how important it is for researchers to pay attention to any intervention process to determine the effects on the subjects.

### Micro-Ethical Moments During the Research Process

As with all research, ethical consent is sought before engaging subjects in research. Conducting research on children requires not only the consent of parents but also the assent of children. For children to effectively participate in a research project, they need to feel that their voices are being heard and that they are voluntarily assisting in the project. For the purpose of this study, an information pack was distributed to the parents^33^ which contained, amongst other items, a consent form from the parent stating their child could participate in research and an assent form for the children. As the sample population of children was between 4 and 5 years, it was assumed that the children could not read so therefore, the assent form also contained pictures explaining that hand swabs would be collected from the children. Each child was then asked to attach a sticker (which was supplied with the form) to the form to indicate assent. There was a parent who indicated that she felt the attaching of the sticker was a coercion of sorts and commented on her own consent form with “Kids want to play with stickers I explained to my child that she didn’t have to place the sticker if she didn’t want to.”

## Discussion

The results of this paper have been documented based on the lived experiences of the researcher during the data collection and intervention phases of an experimental study testing the effects of a hand hygiene intervention on the hand hygiene of preschoolers. The paper aimed to provide a narrative account of the researcher’s experiences and report how involving children in research influenced specific activities and outcomes of the study.

Results reported that children were hesitant or fearful of the Transsystem^®^ Sterile Transport Swab method of collecting bacterial hand data, facilitating the move towards a more child-friendly “bag-wash” collection method. Many children, as paediatric patients, are exposed to healthcare workers at least 31 times from birth to 21 years of age, and commonly report feeling anxious or afraid when in a healthcare-like setting.^18^ Once the researcher explained the purpose of the swabs and the results they were going to test for, their fears were allayed. The uneasiness of the children could possibly be attributed to fear of the unknown,^24^ whereas interacting and providing information gave them a feeling of control and participation towards a meaningful outcome in the study. Additionally, switching to “Bag-wash” samples was found to be more acceptable to the children and perceived by them to be a less “medical” approach, once again highlighting the importance of listening to children’s voices either through their words or non-verbal actions.^2^

This fear of the unknown was further indicated with the hesitance of children to accept male research assistants to help collect hand swab data, as they were familiar with the female researcher. Building a relationship with research participants who are children is an interesting dynamic as there is a moral interdependency with the researcher needing to apply an ethical approach when encountering children, with the uncertainty and unpredictability which can occur.^25^ Children who enjoy good relationships with their teachers tend to learn more easily and are more likely to pay attention if they feel the teacher cares.^26^ The theory that a perceived similarity can improve relationships was adapted by the researcher, to provide a measure of comfort and familiarity to the children.^24^

Listening to children’s voices during research can also be interpreted as observing their actions. The impact of children’s behaviour and actions was shown during the observation phase, where, because of their actions the researcher was able to adjust the observation tool to capture additional data, which, although not relevant to the objectives of the intervention study were seen as a useful tool for educating caregivers on children’s hand-mouth behaviour. Noting the number of times children’s hands or objects were placed in their mouths during specific activities provided insights into children’s classroom behaviour and the impact this behaviour could have on the possible transmission of bacteria through a hand to mouth route, as fomites are known to be a source of bacteria, where 40% of fomites collected at a study in a Nigerian preschool having isolated *Bacillus sp*.^27^

A major part of the intervention was demonstrating correct handwashing procedures and allowing children to mimic the actions. The WHO hand washing method incorporates the use of soap which is known to decrease the odds of diarrhoeal episodes in children and households^10^ reducing diarrhoeal risk by up to 47%.^28^ The method uses a flow phenomenon through hand movements, which not only emulsifies the lipid content on hands allowing the soap to envelope the contents, but through the mechanics of hand washing can slough cells and bacteria from the hand’s surfaces.^29^ Demonstrating this method to preschoolers has been used in several intervention studies,^28^ however, these studies do not record whether children experience difficulties in following the steps in the method. The method requires one to apply soap, rub palms together and then rub the backs of the hands and between the fingers by splaying the fingers and placing the palm of one hand onto the back of the other hand and using the top hand’s palm and fingers to rub the back and between the fingers of the bottom hand before repeating the process with the other hand.^21^

This is a widely used method of handwashing; however, it should not be assumed that all persons can perform the procedure with the same amount of dexterity. The researcher observed that 18.5% of children who participated in the study were not able to perform the actions without assistance. Building a rapport with the children, gaining their trust and having a relationship based on familiarity allowed to researcher to correct these children’s hand movements without creating a situation where the child’s counter-will instinct was activated. Children can resist interventions for various reasons, including counter-will, lack of attachment, misunderstanding, lack of perceived need, emotional regulation, attention seeking and external factors.^30^ MacNamara^30^ states that children naturally have a counter-will instinct, which manifests as walking slowly when asked to walk faster or doing the opposite of what they are told. Acknowledging these factors and making provision for them whilst conducting an intervention or collecting data from young children can improve the study outcomes.

The most rewarding part of the intervention process was that, although it is not possible to document and quantify this, the intervention could be seen as a success from a retention of knowledge perspective by the actions of the children. The study required the researcher, as the principal data collector, to visit the participating preschools throughout the duration of the study (February-December 2019). The intervention took place between July and September 2019, depending on the availability of each school with post-intervention data collected thereafter. During this period, as the researcher entered the classroom, children would randomly hold up their hands and demonstrate the hand-washing actions, indicating that through the interactive and entertaining intervention, they had been able to learn and retain handwashing knowledge. This approach to teaching children to wash their hands can be directly attributed to learning-through-play, which is described as engaging children in meaningful, joyful, repetitive and socially interactive experiences to develop a skill holistically.^31^ Preschools that were exposed to the intervention undertook to continue with the hand hygiene practices as part of their daily activities. These practices can be reinforced during the routine inspections of environmental health practitioners and other health officials. The intervention, specifically the use of Glo-germ as a hand hygiene awareness tool, has been used successfully in other studies, thereby ensuring its transferability.^14^ The researchers have used similar methods for hand washing education in communities and schools as an effective method for teaching hand washing as a result of the success of the intervention undertaken in this study.

Children unintentionally applied Halsted’s model of “See one, do one, teach one” often used during surgical training to educate surgical interns, where learning retention was reported to be greater when training was multisensory and linked to an activity.^32^ The concept of “See one, do one, teach one” is explained by demonstrating an activity to a person, allowing them to conduct the activity and then, once mastered, teaching someone else to do the same activity. Part of the intervention included sending hand hygiene messages to parents via WhatsApp. This effectively opened communication between the researcher and the parents, who reported that their children came home after the intervention and showed their parents how to wash hands in the same way they learned during the intervention.^33^ The study reported an improvement in both parent’s HH behaviours and a decrease in families’ HH-related illnesses, which could potentially be linked to the teaching and learning that took place in the home. Using children to convey a message or to teach others a specific technique by applying the “See one, do one, teach one” method could be incorporated into an intervention programme.

During the study, several micro-ethical moments were presented, namely changing the method of bacterial hand data collection, as well as ensuring that only female research assistants were deployed to collect this. As previously described, during hand-swabbing where children were hesitant to be swabbed by male students whom they had not seen before, the researcher needed to reflect and weigh up the benefits of students participating in experiential learning experiences, or children being made uncomfortable. Using the principle that “a child’s wishes should be paramount,”^34^ the researcher opted to remove the students from the data collection process.

The intervention was an integral part of the study and the study participants needed to participate. However, as this was also seen as a fun and entertaining activity there was a need for further ethical reflection to determine whether all children in the class should participate in the intervention or only those that were part of the study. The ethical consent included that owners of the preschool and preschool caregivers consented to the research taking place, it was therefore decided that this activity as well as the classroom observations, could be applied to all children. As hand washing is part of daily life and correct hand washing can improve health outcomes^6^ teaching all children in the class how to wash hands did not impact on the ethical responsibility of the researcher and also provided a sense of inclusion, therefore not influencing or unbalancing the existing relationship dynamics in each class.^36^

The ethical considerations when conducting studies with children as research participants, and in this case, children under the age of 5 years, requires that ethical consent, as well as the child’s assent, be of the highest standard. There is a fine line between informing children to obtain their assent to participate, and enticing children to participate. There is also a very real possibility that, as parents are gatekeepers to their children, they could either coerce them into participating or dissuade them from the study.^34^ Parents sometimes feel that conducting HH interventions on children could potentially create a situation where children become obsessed with hand washing, with a fear of them becoming germophobic, as well as believing that young children do not have enough HH awareness, thereby accepting that young children will contract HH-related illnesses as a part of their development.^35^ Studies sometimes make use of verbal assent from children,^31^ but this could elicit a “yes” bias, whereas, although the sticker could be seen as fun and in the case of a single parent as a type of co-erosion, it gave the child a sense of autonomy and control.

Involving children in research can create “micro-ethical” moments throughout the study and researchers need to be cognisant of this, putting the child’s needs and comfort above the need to collect data.^36^ During this research, several micro-ethical moments occurred. Schürer and Jensen^36^ stated that they encountered a micro-ethical moment when children not enrolled in the study asked to participate. Allowing a child to participate would mean that they would be doing so without their parent’s consent, but refusing them would run the risk of upsetting the other participants and possibly having long-term repercussions in the relationship dynamics of the group.^34^ Similarly, during the hand swab/bag-wash data collection phase, children wanted to participate as it seemed like a fun activity and the children didn’t want to miss out on this. The researcher was able to include those children who did not have consent by allowing them to observe the data collection process and explaining the process to everyone as a collective, creating a feeling of inclusion as opposed to exclusion.

## Conclusion

Involving children in research requires a certain amount of reflexivity from the researcher. Listening to children’s voices, adapting to accommodate their concerns and seeing children as valuable resources can assist study outcomes. Maximising the potential asset of a child study population must be done in a sensitive manner, acknowledging the rights and agency of each child and constantly evaluating the ethics of each interaction with the children. Results showed that children effectively played a role in shaping the research process and, through their participation, influenced the quality of the ethical considerations and methodology. The narrative in this study reports that children should not be disregarded and can play a valuable role in shaping the aspects of a study, allowing them to contribute meaningfully. These results can assist in urging researchers to include a measure of reflexivity and flexibility in policies and protocols for studies, especially those involving children. Prospective researchers should be encouraged to take extensive field notes to provide additional information on the results of the data collected. Research protocols may need to be adjusted based on the reactions of the research subjects to obtain successful outcomes, and using the findings made by the researcher in this study can assist other researchers to be motivated to be cognisant and aware of the needs and emotions of their participants, as well as the valuable role they play in shaping data collection tools and the data itself.

### Strengths and Limitations

All data in this study were collected by the researcher (with the exception of the hand swab data), thereby allowing for a single, uniform perspective on all aspects of the study. There is, however, a possibility of researcher bias created by using a single researcher with possible preconceived thoughts and a lack of diverse perspectives, which could be prevented by including more researchers during the data collection phase. A more coordinated, structured method of capturing observations and responses by the researcher should have been implemented. There is an opportunity to include the voice of the researcher and the research participants in studies to provide a greater depth and richness to the data.

## References

[1] Sarkadi A Thell M Fängström K , et al. Are we ready to really hear the voices of those concerned? Lessons learned from listening to and involving children in child and family psychology research. Clin Child Fam Psychol Rev. 2023;26(4):994-1007. 37700107 10.1007/s10567-023-00453-4 PMC10640438

[2] Murray J . Hearing young children’s voices. Int J Early Years Educ. 2019;27(1):1-5.

[3] Metcalfe J Hardie KR Segal JI , et al. Impact of an educational intervention upon the hand hygiene compliance of children. J Hosp Infect. 2013;85(3):220-225. 24080083 10.1016/j.jhin.2013.07.013

[4] Pasupuleti D Sasidharan S Manikutty G Das AM Pankajakshan P Strauss S . Co-designing the embodiment of a minimalist social robot to encourage hand hygiene practises among children in India. Int J Soc Robot. 2023;15(2):345-367. 36778903 10.1007/s12369-023-00969-3 PMC9900201

[5] Wong M Choi K Barak L , et al. Young children’s directed question asking in preschool classrooms. Behav Sci. 2024;14(9):754. 39335969 10.3390/bs14090754 PMC11428670

[6] Khan S Ashraf H Iftikhar S Baig-Ansari N . Impact of hand hygiene intervention on hand washing ability of school-aged children. J Family Med Prim Care. 2021;10(2):642-647. 34041054 10.4103/jfmpc.jfmpc_1906_20 PMC8138401

[7] Schmidtke KA Drinkwater KG . A cross-sectional survey assessing the influence of theoretically informed behavioural factors on hand hygiene across seven countries during the COVID-19 pandemic. BMC Public Health. 2021;21(1):1432. 34289816 10.1186/s12889-021-11491-4 PMC8293513

[8] Wolf J Hubbard S Brauer M , et al. Effectiveness of interventions to improve drinking water, sanitation, and handwashing with soap on risk of diarrhoeal disease in children in low-income and middle-income settings: a systematic review and meta-analysis. Lancet. 2022;400(10345):48-59. 35780792 10.1016/S0140-6736(22)00937-0 PMC9251635

[9] Black RE Perin J Yeung D , et al. Estimated global and regional causes of deaths from diarrhoea in children younger than 5 years during 2000-21: a systematic review and Bayesian multinomial analysis. Lancet Glob Health. 2024;12(6):e919-e928. 10.1016/S2214-109X(24)00078-0 PMC11099298 38648812

[10] Noguchi Y Nonaka D Kounnavong S Kobayashi J . Effects of hand-washing facilities with water and soap on diarrhea incidence among children under five years in lao people’s democratic republic: a cross-sectional study. Int J Environ Res Public Health. 2021;18(2):1-15. 10.3390/ijerph18020687 PMC7829977 33466953

[11] Watson J Amon-Tanoh MA Deola C , et al. Effect of a novel hygiene intervention on older children’s handwashing in a humanitarian setting in Kahda district, Somalia: a cluster-randomised controlled equivalence trial. Int J Hyg Environ Health. 2023;250:114163. 10.1016/j.ijheh.2023.114163 37011505

[12] Lin-Siegler X Dweck CS Cohen GL . Instructional interventions that motivate classroom learning. J Educ Psychol. 2016;108(3):295-299.

[13] Watson J Dreibelbis R Aunger R , et al. Child’s play: harnessing play and curiosity motives to improve child handwashing in a humanitarian setting. Int J Hyg Environ Health. 2019;222(2):177-182. 30219482 10.1016/j.ijheh.2018.09.002

[14] Lary D Calvert A Nerlich B , et al. Improving children’s and their visitors’ hand hygiene compliance. J Infect Prev. 2020;21(2):60-67. 33425018 10.1177/1757177419892065 PMC7754805

[15] Arıkan D Gürarslan Baş N Kurudirek F Baştopcu A Uslu H . The effect of therapeutic clowning on handwashing technique and microbial colonization in preschool children. J Nurs Scholarsh. 2018;50(4):441-450. 29764000 10.1111/jnu.12392

[16] Altun D . Preschoolers’ emergent motivations to learn reading: a grounded theory study. Early Child Educ J. 2019;47(4):427-443.

[17] Thomas C Cockcroft E Jenkins G Liabo K . Working with children and young people in research: supportive practices and pathways to impact. J Child Health Care. 2025;29(1):34-52. 37186542 10.1177/13674935231171451 PMC11874604

[18] Freire K Pope R Jeffrey K Andrews K Nott M Bowman T . Engaging with children and adolescents: a systematic review of participatory methods and approaches in research informing the development of health resources and interventions. Adolesc Res Rev. 2022;7(3):335-354.

[19] Lange SL . The effect of a simple intervention on hand hygiene related diseases in preschools. Doctoral thesis. University of Johannesburg; 2021. Accessed February 2, 2025. http://hdl.handle.net/102000/0002

[20] South Africa. Department of Health. National Norms and Standards Relating to Environmental Health in Terms of National Health Act, 2003 (Act No. 61 of 2003) [Internet]. Government Gazette; 2013. Accessed October 1, 2025. https://www.gov.za/sites/default/files/gcis_document/201409/36849gen943.pdf

[21] WHO. WHO Guidelines on Hand Hygiene in Health Care: First Global Patient Safety Challenge: Clean Care Is Safer Care. WHO; 2010. 23805438

[22] Chakkour M Hammoud Z Farhat S El Roz A Ezzeddine Z Ghssein G . Overview of Proteus mirabilis pathogenicity and virulence. Insights into the role of metals. Front Microbiol. 2024;15:1383618. 10.3389/fmicb.2024.1383618 PMC11026637 38646633

[23] Singh A Yelverton CJ Barnard TG . Rapid quantification of the total viable bacterial population on human hands using flow cytometry with SYBR® Green I. Cytometry B Clin Cytom. 2019;96(5):397-403. 30851153 10.1002/cyto.b.21776

[24] Lerwick JL . Minimizing pediatric healthcare-induced anxiety and trauma. World J Clin Pediatr. 2016;5(2):143-150. 27170924 10.5409/wjcp.v5.i2.143 PMC4857227

[25] Quinones G Rutanen N Lucas Revilla Y . A cultural-historical exploration of relational ethics in research involving children. Learn Cult Soc Interact. 2023;42:100756.

[26] Gehlbach H Brinkworth ME King AM Hsu LM McIntyre J Rogers T . Creating birds of similar feathers: leveraging similarity to improve teacher–student relationships and academic achievement. J Educ Psychol. 2016;108(3):342-352.

[27] Damilola Wilkie E Olufunke A Sotala TT . African Journal of Microbiology research antibiotic susceptibility profile of bacteria isolated from fomites in some day care centres in Ile-Ife. Niger. 2022;16(4):132-139.

[28] Ejemot RI Ehiri JE Meremikwu MM Critchley JA . Cochrane review: hand washing for preventing diarrhoea. Evid Based Child Health. 2009;4(2):893-939. 10.1002/14651858.CD004265.pub2 18254044

[29] Mittal R Ni R Seo JH . The flow physics of COVID-19. J Fluid Mech. 2020;894:F2.

[30] MacNamara D . Praise for Rest, Play, Grow. Aona Books; 2016:241-242.

[31] Parker R Thomsen BS Berry A . Learning through play at school – a framework for policy and practice. Front Educ. 2022;7:751801.

[32] Kotsis SV Chung KC . Application of the “see one, do one, teach one” concept in surgical training. Plast Reconstr Surg. 2013;131(5):1194-1201. 23629100 10.1097/PRS.0b013e318287a0b3 PMC4785880

[33] Lange S Barnard TG Naicker N . The effect of a hand hygiene intervention on the behaviour, practices and health of parents of preschool children in South Africa. Perspect Public Health. 2022;142(6):338-346. 36128937 10.1177/17579139221123404 PMC9720708

[34] Nixon LS Hudson N Culley L , et al. Key considerations when involving children in health intervention design: reflections on working in partnership with South Asian children in the UK on a tailored management and intervention for asthma (MIA) study. Res Involvement Engagem. 2022;8(1):9. 10.1186/s40900-022-00342-0 PMC8883750 35227322

[35] Biezen R Grando D Mazza D Brijnath B . Visibility and transmission: complexities around promoting hand hygiene in young children – a qualitative study. BMC Public Health. 2019;19(1):1-8. 30975108 10.1186/s12889-019-6729-x PMC6460784

[36] Schürer M Jensen J . Micro-ethical moments as a part of involving children in research. Des Learn. 2022;14(1):179-189.

